# Synthesis, Characterization and Thermal Properties of Poly(ethylene oxide), PEO, Polymacromonomers via Anionic and Ring Opening Metathesis Polymerization

**DOI:** 10.3390/polym9040145

**Published:** 2017-04-21

**Authors:** George V. Theodosopoulos, Christos Zisis, Georgios Charalambidis, Vasilis Nikolaou, Athanassios G. Coutsolelos, Marinos Pitsikalis

**Affiliations:** 1 Laboratory of Industrial Chemistry, Department of Chemistry, National and Kapodistrian University of Athens, Panepistimiopolis Zografou, 15771 Athens, Greece; scimaster1821@gmail.com (G.V.T.); chriszisis1994@gmail.com (C.Z.); 2 Laboratory of Bioinorganic Chemistry, Department of Chemistry, University of Crete, Voutes Campus, 71003 Heraklion, Greece; gxaral@chemistry.uoc.gr (G.C.); nikolaouvasilis13@hotmail.com (V.N.); acoutsol@uoc.gr (A.G.C.)

**Keywords:** anionic polymerization, ring opening metathesis polymerization (ROMP), polymacromonomers, macromonomers, poly(ethylene oxide), norbornene, Grubbs catalyst

## Abstract

Branched polymers are a valuable class of polymeric materials. In the present study, anionic polymerization techniques were employed for the synthesis of low molecular weight poly(ethylene oxide) (PEO) macromonomers bearing norbornenyl end groups. The macromonomers were characterized by SEC, MALDI-TOF and NMR spectroscopy. Subsequent ring opening metathesis polymerization (ROMP) of the macromonomers using ruthenium catalysts (Grubbs catalysts of the 1st, 2nd and 3rd generations) afforded the corresponding polymacromonomers. The effects of the macromonomer molecular weight, the type of the catalyst, the nature of the solvent, the monomer concentration and the polymerization temperature on the molecular characteristics of the branched polymers were examined in detail. The crystallization behavior of the macromonomers and the corresponding polymacromonomers were studied by Differential Scanning Calorimetry (DSC). The thermal stability and the kinetics of the thermal decomposition of the samples were also studied by Thermogravimetric Analysis (TGA). The activation energies of the thermal decomposition were analyzed using the Ozawa–Flynn–Wall and Kissinger methodologies.

## 1. Introduction

Great effort has been devoted to the synthesis of multifunctional and architecturally demanding polymers and the study of their properties [1,2,3,4,5,6,7,8]. Through specific architectural tailoring, novel polymeric materials arise having different properties compared to their linear analogs [9,10,11,12]. Branched polymers, composed of polymeric chains regularly spaced along a polymeric backbone, have attracted much attention mainly due to their unique possibility of tailoring materials properties through suitable selection of the polymer backbone and the graft chains, thus leading to a large array of applications [13,14]. Out of all the types of branched polymers, polymacromonomers, also referred to as bottlebrushes, in which the side chains are densely distributed along the polymeric backbone, have lately been the subject of intense research [15,16,17]. Due to the crowding arrangement of the side chains, they are stretched away from the backbone forming brush like or worm like conformations. These complex structures can be synthesized through various synthetic approaches including the grafting through, grafting from and grafting to methodologies [18]. “Grafting to” and “grafting from” depict synthetic strategies that involve chemical modifications on the linear polymer backbone. Grafting to proceeds through polymer coupling on the backbone and grafting from proceeds through the growth of a polymer chain from the backbone of a linear multi-initiator. The aforementioned grafting to methodology suffers from limited and inconsistent grafting densities, whereas grafting from approach leads to high decrease of branching but also to high molecular weight and structural heterogeneity. The grafting through method describes a polymerization process using macromonomers as the polymerizing unit. Macromonomers are oligomeric or polymeric chains bearing polymerizable end-groups. Homopolymerization of macromonomers leads to densely grafted structures. This method holds the advantage of uniform grafting density and well-defined side chains; however, propagation takes place under high steric hindrance [15,16]. A huge variety of polymacromonomers has been synthesized by controlled/living polymerization methods, such as anionic, cationic, nitroxide-mediated radical (NMP), atom transfer radical (ATRP), reversible addition-fragmentation chain transfer (RAFT), coordination, ring opening metathesis (ROMP) and ring opening polymerization (ROP) [15,16,17,18].

Poly(ethylene oxide) (PEO) is a neutral, non-toxic, biocompatible and water soluble polymer which has found numerous applications, such as in conductive composites with carbon black, cosmetology (skin creams, emulsions, personal lubricants), gene therapy, pharmaceutical products, etc. [19,20,21,22,23,24]. PEO-based graft copolymers have been investigated for their wide range of promising abilities, to enhance their favorable properties and tailor their capabilities [22,23,24]. These materials have found applications in nanotechnology, lithium batteries, elastomer fabrication, drug delivery systems [20,25,26,27,28,29] and biomedical implants [30,31]. 

Norbornene is a bicyclic compound with a high ring strain, which allows it to proceed in ROMP reaction in a robust and rapid manner [32,33,34]. Polynorbornene (PNBE) is non-cytotoxic, and thus can be used in biomedical applications. It can also be used in the rubber industry for anti-vibration (rail, building, and industry), anti-impact (personal protective equipment, shoe parts, and bumpers) and grip improvement (toy tires, racing tires, transmission systems, transports systems for copiers, feeders, etc.) [35,36,37]. Norbornene is our metathetic moiety of choice for the preparation of α-NBE-PEO macromonomers.

Herein, we describe the synthesis of PEO bottlebrushes, varying in branch size as well as backbone length. The synthesis of these structures was realized through the combination of two different polymerization techniques, those of anionic polymerization and ring opening metathesis polymerization (ROMP). Adopting the grafting through method, the synthetic process involved the use of a PEO macromonomer functionalized with a norbornene group on one end. The macromonomers were subjected to ROMP using the well-defined, ruthenium-based, Grubbs catalysts resulting in the desired brush-like structures with branches comprised of PEO homopolymers and a backbone of a polynorbornene chain. The crystallization behavior of these materials, the thermal stability and the kinetics of thermal decomposition were also studied by Differential Scanning Calorimetry (DSC) and Thermogravimetric Analysis (TGA), respectively.

## 2. Materials and Methods

### 2.1. Materials

All reagents were purchased from Sigma-Aldrich (St. Louis, MO, USA) and used as received, unless described otherwise. Tetrahydrofurane (THF, Merck, Kenilworth, NJ, USA) and Toluene (Sigma, Kawasaki, Japan) were purified according to the standards of anionic polymerization high vacuum techniques described elsewhere [38]. Methanol was stirred overnight in the presence of a small amount of anhydrous magnesium sulfate (MgSO_4_) and then fractionally distilled into ampoules under vacuum. Dichloromethane (CH_2_Cl_2_) was stirred overnight in the presence of a small amount of calcium hydride (CaH_2_) and then fractionally distilled in a flame dried flask containing 3 Å molecular sieves. Dichlorobenzene (DCBz) was stirred overnight in the presence of a small amount of CaH_2_ and then fractionally distilled in a flame dried flask containing pieces of sodium. 5-Norbornene-2-methanol (Sigma-Aldrich) was used as provided. Ethylene oxide monomer was purified according to the standards of anionic polymerization [38]. Grubbs 1st and 2nd generation type catalysts (Sigma-Aldrich) were used as were received. Grubbs 3rd generation type catalyst was prepared following a previously described method [39].

### 2.2. Synthesis of the Norbornenyl Oxyanion Initiator

**Scheme 1:**
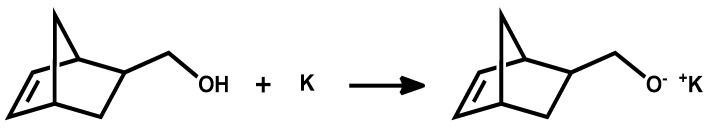
Synthetic route for the preparation of the norbornenyl oxyanion initiator.

The synthesis of the macromonomers was realized via anionic polymerization. Prior to the polymerization process, the preparation of a norbornenyl oxyanion initiator was necessary (Scheme 1). For the purpose of synthesis, 1 mL (1.0270 g, 8.3 mmol) of commercially available 5-norbornene-2-methanol was injected into a glass ampoule, degassed under vacuum and dissolved with 5 mL of pure THF, which were distilled into the ampoule from the high vacuum line [38]. After the distillation the ampoule was degassed and flame-sealed from the high vacuum line. The ampoule containing the alcohol solution was then connected to another specially designed glass apparatus, which is shown in the Supporting Information Section (Figure S1). The apparatus was attached to the vacuum line and, following previously mentioned techniques [40], potassium metal was properly distilled into the flask of the apparatus forming the desired potassium mirror. The apparatus was then sealed under vacuum and the break-seal of the alcohol ampoule was ruptured releasing the alcohol solution in the flask covered with the potassium mirror. The solution was stirred for 3 days and degassed after the first 24 h. The solution containing the oxyanion was then filtered under vacuum through two, P2 type, glass filters and isolated in a flask equipped with a break seal. 

### 2.3. Synthesis of the PEO Macromonomers

**Scheme 2:**
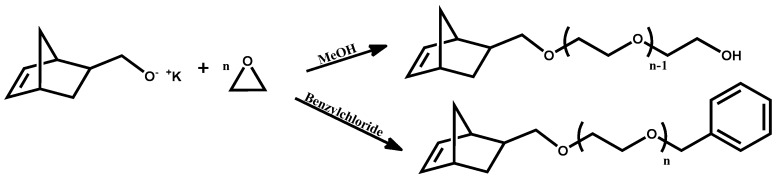
Polymerization of ethylene oxide with the norbornenyl oxyanion initiator and two different terminating agents.

Ethylene oxide was polymerized under high-vacuum in THF using break-seal techniques, and the norbornenyl oxyanion as the initiator. The polymerization reaction (Scheme 2) was performed in a custom-made glass reactor equipped with ampoules containing the oxyanion initiator (8.27 mmol), methanol or benzyl chloride for termination purposes (~2 mL, an excess over the stoichiometric amount) and ethylene oxide. The quantity of ethylene oxide employed, differed according to the desired final molecular weight of the macromonomer (Table 1). In all cases, the same amount of the initiator was used and the polymerization reaction was conducted at concentrations between 5% and 10% *w*/*v*. All polymerization reactions led to the desired molecular weights and to quantitative yields. Initially, the ampoule containing the initiator was introduced by rupturing the appropriate break-seal in the reactor. The temperature of the reaction solution was then raised to 40 °C and then the break seal of the ampoule containing the monomer was carefully ruptured. Prudence is necessary since the gassy nature of the monomer EO might prove hazardous, hence prior to smashing the seal, the monomer was chilled using liquid nitrogen. After rupturing the break-seal of the monomer ampoule EO was slowly and cautiously liquefied. After the complete addition of the monomer the reactor was left in the water bath (40 °C) for 2–3 days depending on the molecular weight. After the completion of the polymerization the terminating agent (MeOH, or benzyl chloride) was added by breaking the break-seal of the appropriate ampoule. The solution was then left to react for 30 min. Finally, the polymer was precipitated in petroleum ether, filtered and dried under vacuum for 24 h.

### 2.4. Synthesis of PEO Polymacromonomers

**Scheme 3:**
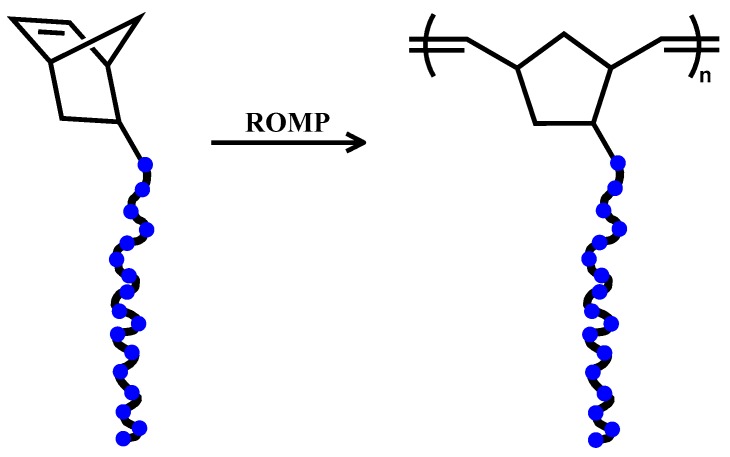
Synthesis of PEO polymacromonomers.

As previously mentioned, the preparation of the polymacromonomers involved the use of ruthenium Grubbs type catalysts. The ROMP process was conducted under inert argon atmosphere, either in a glove box or on an argon-vacuum line according to principles mentioned elsewhere [38,39,40]. Grubbs 1st, 2nd and 3rd generation type catalysts were employed (Scheme 3). In all cases, the monomer concentration of the polymerization medium varied between 250 mg/mL and 125 mg/mL, depending on the targeted molecular weight (larger molecular weights require lower initiator concentrations). ROMP reactions were carried out using 1 g of macromonomer in most cases and similar concentrations. Describing the ROMP reaction, initially 1 g of macromonomer was dissolved in the polymerization medium (THF, CH_2_Cl_2_, DCBz, or Toluene). The desired type of Grubbs catalyst was then dissolved in THF or CH_2_Cl_2_ (0.5 mg/mL) under stirring. After complete dissolution, employing the conventional method, the desired volume of the catalyst solution was added in a vial or a Schlenk flask already containing the macromonomer solution. Employing the seeding process, the catalyst solution was initially added in a vial or a Schlenk flask followed by the addition of a small amount of the macromonomer solution. After 10–15 s, the remaining macromonomer solution was added in the reaction mixture for the completion of the polymerization. In both cases, the polymerization was allowed to proceed for 30 min to 4 h, depending on the targeted molecular weight. Finally, the catalyst was quenched with the addition of a few droplets of ethyl vinyl ether, and the bottlebrush was precipitated in a non-solvent (diethyl ether or petroleum ether), filtered, washed with diethyl ether and vacuum dried. Residual amounts of the catalyst were removed via the use of a soxhlet apparatus and diethyl ether as the refluxing solvent.

### 2.5. Characterization

SEC experiments were conducted at 40 °C using a modular instrument consisting of a Waters Model 510 pump, a Waters Model U6K sample injector, a Waters Model 401 differential refractometer, a Waters Model 486 UV spectrophotometer (Waters Corp., Milford, MA, USA), and a set of 4 μ-Styragel columns with a continuous porosity range from 10^6^ to 10^3^ Å. The columns were housed in an oven thermostatted at 40 °C. THF was the carrier solvent at a flow rate of 1 mL/min. The system was calibrated with seven PS standards having MWs between 1000 and 900,000 g·mol^−1^.

Nuclear magnetic resonance (NMR) spectra were recorded in chloroform-d and deuterated dimethylsulfoxide DMSO-d6 at 25 °C with a Varian Unity Plus 300/54 NMR spectrometer and Liquid State Varian Mercury Vx 300 MHz spectrometer (Varian Associates Inc., Palo Alto, CA, USA). 

Mass spectra were obtained on a Bruker UltrafleXtreme matrix assisted laser desorption ionization time-of-flight (MALDI-TOF) spectrometer (Bruker Corp., Madison, WI, USA) using 2,5-Dihydroxybenzoic acid (DHB) as matrix. 

The glass-transition temperatures were obtained by differential scanning calorimetry (DSC) using a 2910 modulated DSC model from TA instruments (New Castle, DE, USA). The samples were heated or cooled at a rate of 10 °C/min. The second heating results were obtained in all cases. The thermal stability of the copolymers and the kinetics of their thermal decompostion was studied by thermogravimetric analysis (TGA) employing a Q50 TGA model from TA instruments. The samples were heated from ambient temperatures up to 700 °C in a 60 mL/min flow of N_2_ at heating rates of 3, 5, 7, 10, 15 and 20 °C/min.

## 3. Results and Discussion

### 3.1. Synthesis of PEO Macromonomer

The macromonomers were synthesized employing a functional norbornenyl oxyanionic initiator. This method holds the benefit of ensuring functionalization on the polymer chain end. In addition, through the use of a suitable electrophilic terminating agent, there is an ability to introduce functionality on the ω-chain end as well, thus finally creating α, ω bifunctional telechelic polymers. 

The synthesis of macromonomers through modification of the PEO’s end-group employing post-polymerization reactions is not always the desired method because it is difficult to achieve quantitative functionalization reactions. Finally, the product is a macromonomer contaminated with amounts of non-functionalized PEO chains. In order to force the post-polymerization reaction to completion, special reagents in a large excess are required. This excess has to be removed before proceeding further with the synthesis of the bottlebrushes, thus adding more steps in the synthesis of the desired products. 

The preparation of PEO macromonomers using a similar methodology (oxyanionic polymerization) has been previously presented [41,42,43,44]. However, the synthetic method reported involved the in-situ creation of the norbornenyl oxyanion initiator by titrating a norbornenol solution with a triphenyl methyl potassium ((Ph)_3_C^−^^+^K) solution. This method, although experimentally more convenient, cannot be easily performed under high vacuum. Moreover, the titration process ceases after the slight presence of the characteristic red color of (Ph)_3_C^−^^+^K solution. The excess (Ph)_3_C^−^^+^K acts as initiator for ethylene oxide thus generating macromolecules without a functional norbornenyl end-groups. 

The use of commercially available ω-hydroxy-PEOs in PEO macromonomer is not advised, since these compounds are known to contain traces of dihydroxy species as well. A method involving the use of commercially available PEO-NH_2_ covalently bonded on a norbornene moiety has also been reported [45]. However, several reaction steps are required for the synthesis, in addition to the presence of any traces of impurities finally leading to rather low yields and undesired molecular characteristics. Our choice to synthesize the norbornelyl oxyanion initiator prior to the polymerization process ensures 100% functionality of the macromonomers. Termination of the macromonomers was mainly achieved, with the addition of methanol, transforming the alcoholates to the corresponding hydroxyl groups. Benzyl chloride was also used to deactivate the anionic centers. The two different types of macromonomers having either ω-hydroxy or ω-benzyl groups were prepared in order to compare the corresponding ROMP reactions between the two different types of macromonomers [41]. The presence of the ω-hydroxy group offers the possibility to conduct further chemistry leading to more complex macromonomer structures. However, it has been reported [46,47,48,49,50] that functional groups such as primary alcohols may react with Grubbs type ruthenium catalysts causing decomposition of the catalyst to monohydride species. Therefore, benzyl terminated macromonomers were also prepared in order to avoid this problem and achieve a better control over the molecular characteristics of the polymacromonomers. 

**Figure 1:**
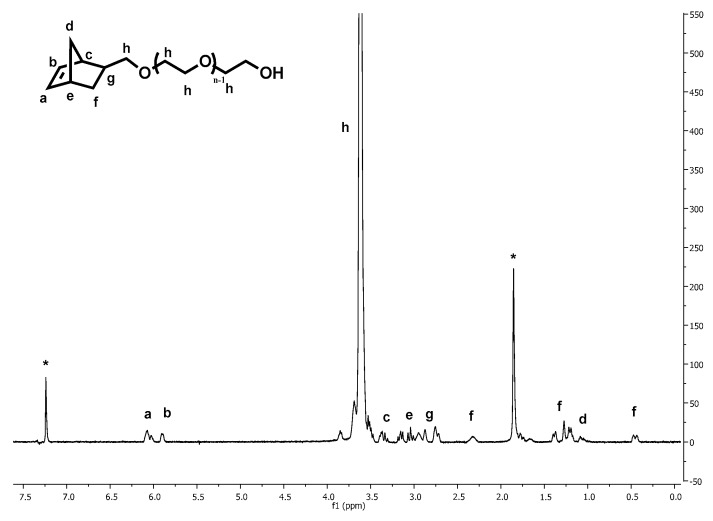
^1^H-NMR spectrum of PEO 1k macromonomer in CDCl_3_.

**Figure 2:**
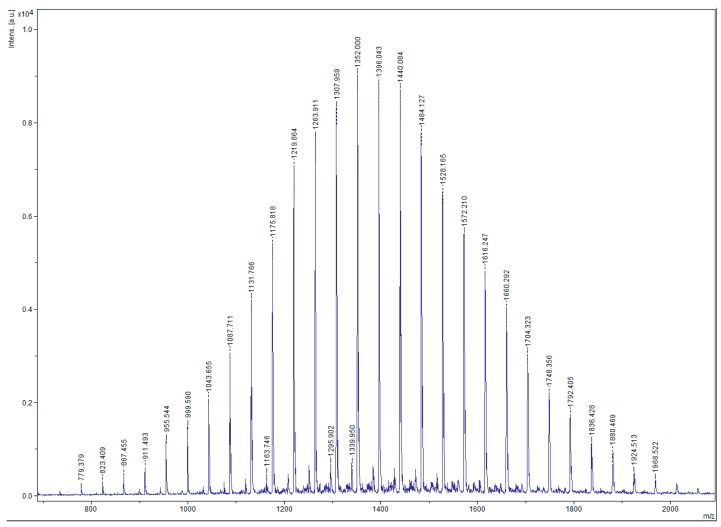
MALDI TOF-MS spectrum of PEO 1k macromonomer.

**Figure 3:**
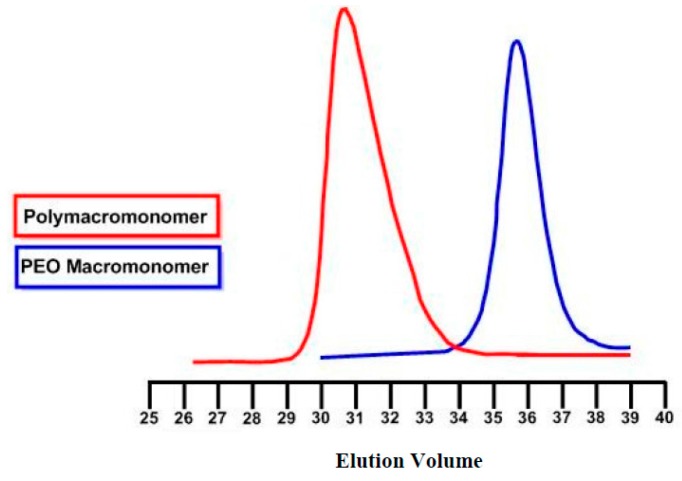
SEC traces of PEO 1k macromonomer and polymacromonomer having *M*_w_ = 25,000, *M*_w_/*M*_n_ = 1.18).

The macromonomer synthesis was conducted in dilute THF solutions at 40 °C. Since the preparation of the initiator involved the use of potassium, a known pyrophoric substance, judicious planning and extreme caution is necessary before handling the metal. Immediately after the addition of the alcohol solution to the potassium, mirror bubbling and a quick decay of the mirror are visible. To achieve quantitative yields, degassing of the solution is necessary. This procedure was applied twice before the termination of the living polymer. Precipitation was realized in chilled diethyl ether, under constant stirring; before pouring the solution in the non-solvent part of the solvent was removed, via evaporation, for better precipitation. After that the macromonomers were filtered, washed with diethyl ether and freeze dried with benzene, in order to remove moister traces from the polymers. This final process is necessary to provide dry samples suitable in the latter step for ROMP reaction. The polymers were then stored and weighted in a glove box. In all cases, the mass of the macromonomers indicated quantitative polymerization yields and characterization with NMR (Figure 1), MALDI TOF-MS (Figure 2) and SEC (Figure 3, Figures S2 and S3) ensured norbornene group functionality and agreement with the targeted molecular weight. The molecular weights of the samples are given in Table 1.

The α-functionality of the macromonomers was confirmed from the NMR spectra. The intensity of the characteristic peaks of the protons (*I*_1_) of the norbornene double bond (δ 5.8–6.2 ppm) when integrated, compared with the intensity of the peak corresponding to the protons (*I*_2_) of the etheric carbons of PEO (δ 3.5–3.7 ppm), were found in close agreement with the equation *I*_2_ = 2$I_{1}\;\times \bar{\;DP}$.

The degree of polymerization was calculated through the MALDI TOF-MS spectra and in all cases was found close to the stoichiometric values. The $\bar{DP}$ calculated from the $\bar{M_{w}}$, extracted from the SEC chromatograms were also close to the absolute values of MALDI TOF-MS.

MALDI TOF-MS was carried out using DHB (2,5-Dihydroxybenzoic acid) and gave better spectra results in the absence of added ionizer. All data confirm that well defined macromonomers with narrow molecular weight distributions are obtained through this oxyanionic polymerization.

### 3.2. Synthesis of PEO Polymacromonomers via ROMP

The synthesis of the PEO macromonomers with the desired molecular characteristics was no trivial task. Other than the possible decomposition of the catalyst to monohydride species from the ω-hydroxyl groups, mentioned previously, the unpaired electors of the oxygen atoms in each repeated monomer unit may also interact with unoccupied *d*-orbitals of the ruthenium metal of the catalyst, thus hindering the ROMP process. After the addition of the catalyst to the monomer solution, the solution color changes initially from green (G 3rd), violet red (G 2nd) or purple (G 1st), depending on the catalyst, to orange, and within a few seconds into red to dark red, indicating the possible interaction of the oxygen atoms with the catalyst transition metal [41]. There is no great difference between the ω-hydroxyl and the ω-benzyl macromonomers, especially for the higher molecular weight macromonomers, where the contribution of the end-group is minimized. After ROMP completion the catalyst is rendered unreactive with the addition of a few drops of ethyl vinyl ether. A color change is observed reduced to light yellow. The solution is then added drop wise into a suitable non-solvent (depending on the solvent used) under constant, vigorous, stir. The polymacromonomer is then filtered, washed with diethyl ether, dried and characterized. 

All three generations of the well-defined Grubbs catalysts were used in various solvents and temperatures, in an attempt to define the optimum polymerization conditions. Grubbs 1st generation catalyst demonstrated poor results with low polymerization yields, broad molecular weight distributions (even bimodal peaks) and non-reproducible molecular weights. The problem was more pronounced for the higher molecular weight macromonomers. Grubbs 2nd generation gave, in most cases, quantitative yields, however with poor control over the molecular characteristics. Bimodal distributions were a common result and only when toluene was used as the solvent monomodal distributions were realized. Much better results, as expected, were obtained with the use of Grubbs 3rd generation catalyst. Quantitative yields were obtained, however reproducible results and monomodal distributions were difficult to acquire in sequential ROMP reaction, showing no dependence on solvent nature or monomer concentration. 

The addition process was proved to be of great significance; specifically ROMP reactions carried out through the seeding process gave reproducible results and monomodal distributions. According to this procedure, a small amount of the macromonomer (three molar excess over the catalyst) is initially added to the catalyst solution and after 10–15 s the remaining amount of macromonomer is added and left for polymerization. With this methodology the initiation step is technically separated from the propagation step. The initial addition of the small amount of macromonomer assures that all the catalyst species are activated at the same time and that they are ready to promote the propagation step, upon the subsequent addition of the remaining quantity of macromonomer, with the same rate leading to uniform polymerization reaction. Molecular characteristics of polymacromonomers prepared by the conventional addition of the catalyst to the macromonomer solution are given in the Supporting Information Section (Table S1), whereas those prepared by the seeding methodology are shown in Table 2. A characteristic example monitoring the synthesis of a PEO polymacromonomer with the seeding procedure is given in Figure 3. Small tailing effects were observed in SEC traces indicating the presence of chemical heterogeneity in the samples, i.e., polymacromonomers with different number of side chains. However, this is not so pronounced taking into account the rather low molecular weight distribution of the bottlebrushes. 

Overall, the synthesis of the PEO polymacromonomers is accompanied by many synthetic challenges. Against all expectations the key factor was neither the solvent nor the monomer concentration but the addition method. The seeding process ensures reproducible results and the optimum molecular characteristics. Nevertheless, all factors have minor effects on the ROMP reactions. From our observations, the primary factors are the following:

• *Molecular weight dependency*

By increasing the length of the macromonomer chain, a decrease over polymerization control was observed. Bimodal distributions were obtained and, in spite of the presence of the norbornene functional group, quantitative yields were not received. This was attributed to coordination phenomena that occur between the electron rich oxygen atoms of the repeating units and the unoccupied *d*-orbitals of the catalyst metal. Reproducibility was also a difficult factor to ensure, probably due to the presence of such phenomena. Quantitative yields and monomodal distributions were constantly obtained for macromonomers with molecular weights up to 3 kDa. Polymacromonomers with branch sizes up to 5 kDa were produced, however quantitative yields and monomodal distributions were difficult or impossible to achieve. It was found that repeatable results were only possible when the seeding process was followed. For macromonomers with molecular weights <3 kDa repeatable results, with decreased polydispersity, were obtained via seeding polymerization. For macromonomers with molecular weights >3 kDa improved results in polydispersity and reaction yields, were observed. The declining results, as the molecular weight of the macromonomers increases, is reasonable since the “density” of the oxygen atoms (possible complexing factors) increases. In other words, keeping in mind that the macromonomer, in solution, adopts a bundle like entangled formation, the coordination of the ruthenium catalyst with the targeted norbornene group is confronted with more oxygen atoms in each chain, as well as the solution in general.

• *Catalyst dependency*

Grubbs 1st generation gave satisfactory and repeating results for macromonomers up to 2 kDa. However, for molecular weight higher than 2 kDa bimodal distributions and non-quantitative yields were acquired. Grubbs 2nd generation, being more tolerant to functional groups gave in all cases quantitative yields, however did not give reproducible results. Grubbs 3rd generation catalyst, gave improved and in general much better results; combining the two major qualities of the other two catalysts, fast initiation rate compared to propagation (G 3rd is 1000 times faster compared to G 1st) and functional group tolerance (G 2nd had better functional group tolerance but a small difference between initiation and propagation rates; leading to broad molecular weight distributions).

In comparative experiments between G 1st and G 3rd catalysts using the same amounts of the same macromonomer, results showed that G 3rd catalysts produced polymacromonomers in better agreement with the equation *M*_w_ *= mass of macromonomer in g/moles of catalyst*, even though complete metathesis was achieved in both cases. G 1st generation catalyst gave larger molecular weights compared to G 3rd. This result could be attributed to the high reactivity and functional group tolerance, leading to rapid polymerization without any obstructions due to coordination phenomena. 

• *Solvent and concentration dependency*

Various solvents were used in a range of concentrations and in different reaction temperatures. Even though large concentrations are favorable for ROMP reactions (for small cycloolefin monomers), in the case of ROMP using macromonomers was not as crucial. On the contrary, lower concentrations were found to give better results. Polar solvents and specifically solvents with greater dielectric constant gave better initiation to propagation rates. With G 2nd catalyst better results were obtained in toluene resulting, in most cases, to monomodal distributions and quantitative yields. Dichloromethane (DCM) was a most reliable solvent for ROMP, however, for ROMP preformed in higher temperatures, dichlorobenzene (DCBz) was found to be the best choice, having a high boiling point and high dielectric constant. ROMP preformed at higher temperatures, up to 70 °C, gave better results in cases were low yields were obtained, increasing the polymerization yields. This was ascribed to the fact that at higher temperatures coordination phenomena between the catalyst and oxygen of the monomeric units become weaker [51,52].

In general the solvents of choice were THF and DCM, giving polymacromonomers in agreement with the equation *M*_w_ *= g macromonomer/moles of catalyst*. There have been reports in literature [53] stating that the solvents THF and DCM result in poor results in the case of polymacromonomers of high molecular weight, advising the use of 1,2-diethoxyethane. There have also been reports [54] on the use of a mixture of solvents specifically EtOH/CH_2_Cl_2_ in a 65/35 ratio. However, this solvent mixture did not give the best results in our experiments, since the presence of primary alcohols may have a destructive effect causing decomposition of the catalyst to monohydride species [46,47,48,49,50].

### 3.3. Thermal Properties of PEO Polymacromonomers

The thermal properties of the polymacromonomers were also investigated and the samples bearing the optimum molecular characteristics were subjected to Differential Scanning Calorimetry (DSC) measurements.

The number and letter code describing the polymacromonomer samples in Table 3 offer data regarding each macromonomer or polymacromonomer. The first number indicates the molecular weight of the macromonomer used in the ROMP reaction, the second number the molecular weight of the polymacromonomer in thousands, the third number the type of Grubbs catalyst used and the final letters the solvent in which the ROMP reaction was carried out.

Due to the ionic conductivity that PEO presents, there has been much interest in their employment in electrochemical devices. However, PEO aids in ion transportation only in the amorphous phase, and, being a semi-crystalline polymer, presents crystallinity at room temperatures; thus, their use in such applications has been limited. Hence, the design-criterion for PEO-based electrolytes has been focused on the suppression of PEO crystallinity and the increase of the amorphous percentage. Crystallinity (for PEO) is normally increased with molecular weight: up to 1000 Daltons, it remains a viscous liquid with glass transition temperatures below room temperature. Our scope was to study the effect of the total brush and branch molecular weight on crystallinity. For that reason, a series of polymacromonomers differing in branch and backbone size were prepared. During the building process of the polymacromonomers, due to the polymer chain congestion and repulsive forces present, the side chains adopt a rod-like conformation leading to a final cylindrical structure. The backbone of the PEO bottlebrushes consisted of norbornene units. Polynorbornene is a rather flexible chain, judging from their low *T*_g_ value [55,56], thus providing a relative flexibility, overall, to the highly dense structure. 

The tailoring of PEO macromonomers at the significantly dense backbone extensions results in the PEO chains adopting an entropically unfavourable chain conformation, with hydrodynamic volumes much smaller than that of their random coil structures [14,57]. These entropic alterations could possibly have an effect on the crystallinity of the polymers. In other words, the crowding of the PEO chains in the bottlebrush allows them to adopt a stretched, less flexible, rod-like conformation. In this case, the PEO chains have a smaller hydrodynamic volume than that when they had a random coil conformation. 

Analyzing the DSC results for the linear PEO macromonomers, it is observed that both the melting point and the degree of crystallinity increase upon increasing the molecular weight of the macromonomer. This result is quite reasonable in view of the low molecular weight of the macromonomers. On the contrary, polymacromonomers showed much smaller Δ*Η*_M_ values or in other words a substantial reduction of the crystallinity compared to the corresponding macromonomers from which they were synthesized. If the difference in molecular weight between the macromonomers and their polymacromonomers is taken into account then the reduction of crystallinity is even more pronounced. 

The samples 1-15-3, 2-15-3 and 3-15-3 have similar total molecular weights but different number of branches. It is obvious that a decrease of the number of branches leads to an increase of both the crystallinity and the melting point, extending the effect of the macromolecular architecture on the thermal properties of the materials. Similar results are extracted from the comparison of the samples 1-47-1, 2-45-3, and 3-50-3. 

PEO bottlebrushes have been previously synthesized by many groups and their thermal properties have been studied. According to the literature, the crystallinity increases upon increasing the total molecular weight of the PEO bottlebrush (backbone) [58,59,60]. Studying the results by thermal analysis it can be concluded that crystallinity and melting point are both depressed upon increasing the number of the side chains and upon decreasing the molecular weight of each branch. 

### 3.4 Kinetics of the Thermal Decomposition of the PEO Macromonomers and Polymacromonomers

**Figure 4:**
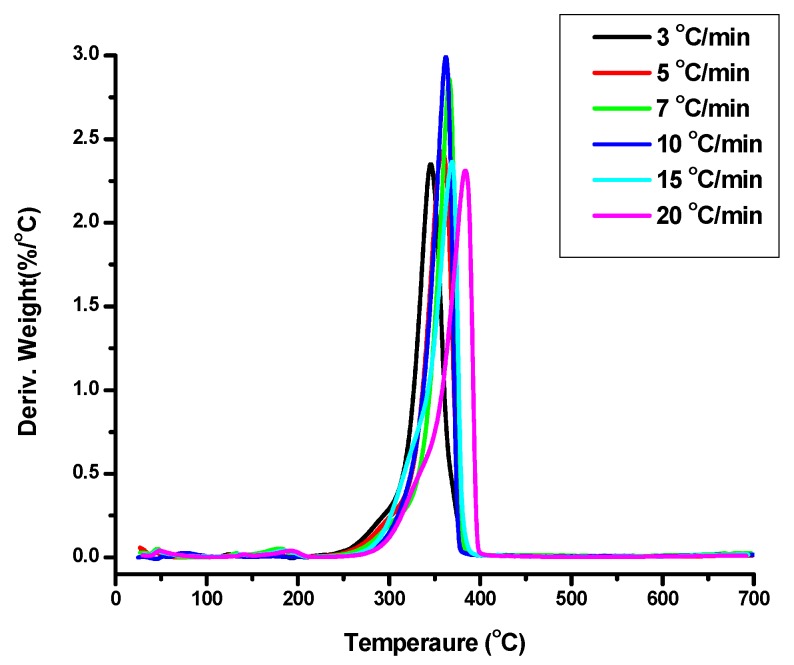
Derivative weight loss with temperature for PEO 1k under different heating rates.

**Figure 5:**
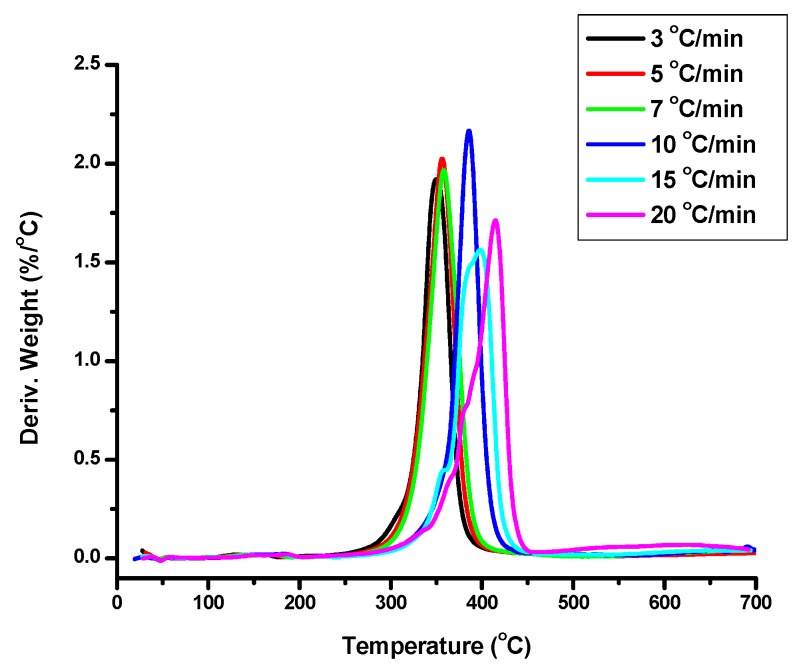
Derivative weight loss with temperature for 1-205-3-THF under different heating rates.

The PEO macromonomers with molecular weights 1k, 2k, 3k and three of the polymacromonomers, namely 1-205-3-THF, 2-45-3-THF and 3-50-3-THF, were thermally degraded at different heating rates under inert atmosphere. The samples 2-45-3-THF and 3-50-3-THF were selected because they have similar total molecular weight but differ in the number of branches and branch molecular weight. Sample 1-205-3-THF was chosen, since it has a very high number of branches. The temperatures where the thermal decomposition is initiated and completed along with the temperatures at the maximum rate of thermal decomposition are provided in Table 4 (PEO macromonomer 1k) and Table 5 (sample 1-205-3-THF) and characteristic thermograms from Differential Thermogravimetry (DTG) are given in Figure 4 and Figure 5. Additional data (Tables S2–S5) and plots (Figures S4 and S5) are given in the Supplementary Materials Section.

The temperature of the thermal decomposition at the peak of DTG (*T*_p_) is similar for the macromonomers PEO 1k and PEO 2k, whereas a slightly higher value is observed for macromonomer PEO 5k. It is obvious that, for these low molecular weight semicrystalline polymers, an increase in molecular weight should result in an increase of the intermolecular interactions leading to higher thermal stability. The difference in molecular weight for samples PEO 1k and PEO 2k is rather low to observe this effect. However, PEO 5k has a substantially higher molecular weight leading to higher *T*_p_ value. For all macromonomers a simple and symmetrical peak is obtained in DTG with a rather short range of decomposition temperatures thus indicating the presence of a relatively simple decomposition mechanism. The residue of the thermal decomposition is lower than 5%. However, an even lower value (less than 3%) is obtained for PEO 5k. This is a manifestation of the effect of the norbornene end groups. It is known that in polynorbornene from ROMP reactions a high residue is obtained after the thermal decomposition in inert atmosphere [61,62]. Therefore, samples PEO 1k and PEO 2k with the higher content in norbornene end groups have higher residues than sample PEO 5k.

By comparing the macromonomers with the polymacromonomers, it is clear that the branched structures show much higher temperatures for the initiation and the completion of the decomposition than the linear ones. Specifically, this increase is up to 50 °C for sample 1-205-3-THF, whereas for samples 2-45-3-THF and 3-50-3-THF is up to 20–30 °C. The increased thermal stability is attributed to the presence of the PNBE backbone of the bottlebrushes, since PNBE is thermally much more stable than PEO. This thermal stability is even more pronounced for sample 1-205-3-THF, since this sample has the highest degree of polymerization and therefore the highest NBE content compared to the other polymacromonomers. This result indicates that the decomposition of the PEO side chains probably starts from the free end polymer groups (hydroxyl groups). Since the chain end is chemically connected to the PNBE backbone, its thermal stability is substantially increased. However, DTG reveals that the *T*_p_ values are not very different compared to the macromonomers, since this value is influenced by the nature of the PEO chains, which is common in both the linear and the branched structures. In contrast to the linear macromonomers, the bottlebrushes show shoulders in DTG, especially at lower temperatures, meaning that the presence of the PNBE backbone makes the mechanism of decomposition of the polymacromonomers more complex. The residue is substantially increased for the bottlbrushes due to the presence of the PNBE backbone. The higher the PNBE content the higher the residue at 700 °C.

Comparing the polymacromonomers, it is concluded that samples 1-205-3-THF and 2-45-3-THF have similar ranges of temperatures for their thermal decomposition. However, sample 3-50-3-THF has increased temperatures of decomposition, which reflects the higher thermal stability of PEO 3k compared to the other macromonomers.

The kinetics of the thermal decomposition of the samples was studied by TGA measurements. The activation energy, *E*_a_, of mass loss upon heating was calculated using both the isoconversional Ozawa–Flynn–Wall (OFW) [63,64] and Kissinger [65,66] methods. The OFW approach is a “model free” method that assumes that the conversion function *F*(α), where α is the conversion, does not change upon altering of the heating rate, β, for all values of α. The OFW method involves the measurement of the temperatures corresponding to fixed values of *α* from experiments at different heating rates β. Therefore, plotting lnβ vs. 1*/T* in the form:

$$\ln\beta \;=\;\ln\;\frac{AE}{R}-\ln F\left(\alpha \right)-\;\frac{E}{RT}$$

This should result in straight lines with slopes directly proportional to the activation energy where *T* is the absolution temperature. *A* is the pre-exponential factor (min^−1^) and *R* is the gas constant (8.314 J/K·mol). If the determined activation energy is the same for the various values of α, then a single-step degradation reaction can be concluded. The OFW method is the most useful method for the kinetic interpretation of thermogravimetric data, obtained from complex processes like the thermal degradation of polymers. This method can be applied without knowing the reaction order. 

The activation energy *E*_a_ was also calculated from plots of the logarithm of the heating rate vs. the inverse of temperature at the maximum reaction rate in constant heating experiments, according to the Kissinger method. The equation for the Kissinger method is the following:

$$\ln\left(\frac{\beta }{T_{p}^{2}}\right)=\ln\frac{AR}{E}+\ln\left[n{\left(1-a_{p}\right)}^{n-1}\right]-\frac{E}{RT_{p}}$$

where $T_{p}$ and $a_{p}$ are the absolute temperature and the conversion at the maximum weight loss and *n* is the reaction order. The *E*_a_ values can be calculated from the slope of the plots of ln(β/*Τ*_p_^2^) vs. 1/*T*_p_. 

**Figure 6:**
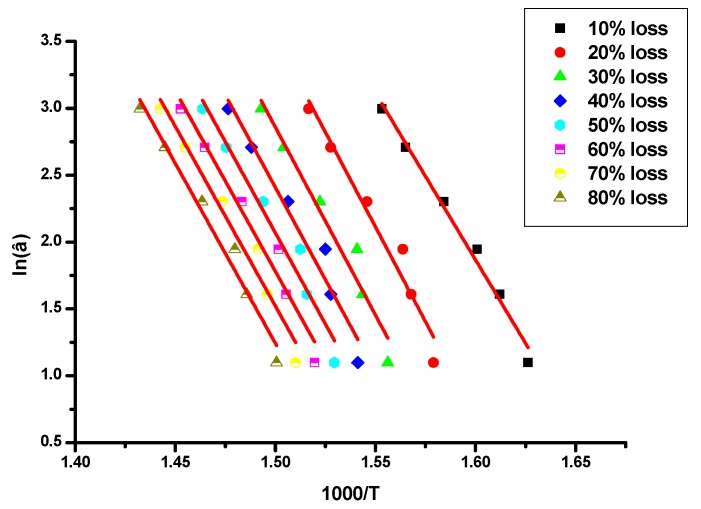
OFW (Ozawa–Flynn–Wall) plots for 3-50-3-THF.

**Figure 7:**
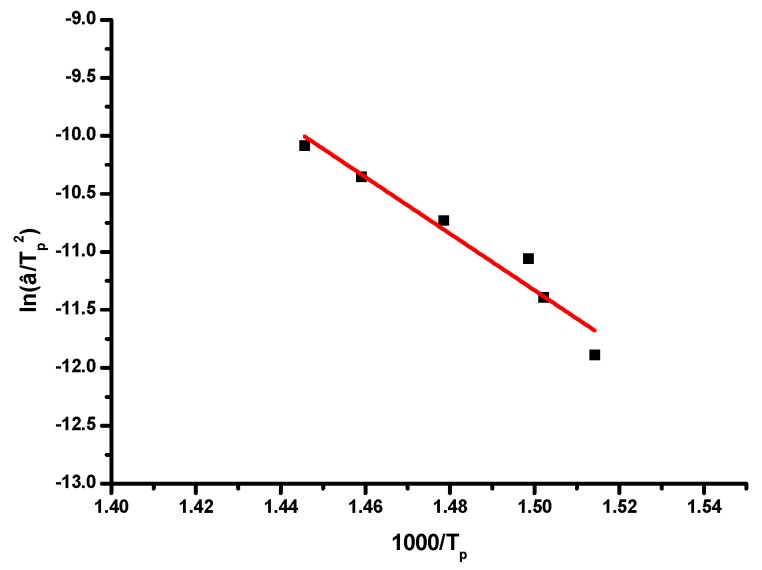
Kissinger plot for 3-50-3-THF.

The activation energy values, *E*_a_, of the samples resulting from the OFW method are displayed in Table 6, whereas characteristic plots are given in Figure 6 and Figure S6. The corresponding results from the Kissinger method are displayed in Table 7 and a characteristic plots are given in Figure 7 and Figure S7. 

The *E*_a_ values for the macromonomers do not vary substantially with the conversion α (OFW method) indicating that the decomposition mechanism is rather simple, in agreement with the DTG results. Upon increasing the molecular weight of the macromonomer the *E*_a_ values are decreased, meaning that a lower energy barrier is required for the thermal decomposition of the higher molecular weight macromonomers. This result reflects the effect of the norbornene end-group in fortifying the thermal stability of the polymers. The lower the molecular weight of the macromonomer, the higher is the effect of the norbornene end-group and thus the higher the thermal stability. Similar conclusions were drawn by DTG. The *E*_a_ values obtained from the Kissinger method are qualitatively in close agreement with those obtained from the OFW method. However, as it is common in the literature, the Kissinger approach leads to lower *E*_a_ values than the OFW approach. 

In the case of the polymacromonomers the *E*_a_ values, according to the OFW analysis, vary slightly with the conversion as a manifestation of the more complex mechanism of thermal decomposition. This variation is more pronounced for sample 1-205-3-THF, obviously due to the higher norbornene content of this sample. However, in contrast with the macromonomers, the *E*_a_ values are decreased upon decreasing the molecular weight of the macromonomer. The higher the number of end groups in the structure, the lower is the energy barrier that is required for the thermal decomposition. Combination of the branch molecular weight and the degree of polymerization of the bottlebrush leads to an increase of end groups in the order: 3-50-3-THF < 2-45-3-THF < 1-205-3-THF.

## 4. Conclusions

A combination of anionic and ring opening metathesis polymerization techniques were used in order to produce poly(ethylene oxide) (PEO) macromonomers and the corresponding polymacromonomers. SEC, MALDI-TOF and NMR spectroscopy were used to characterize these materials. Parameters such as the molecular weight of the macromonomer, the structure of the catalyst, the nature of the solvent, the monomer concentration and the polymerization temperature were examined in order to achieve the best control over the molecular characteristics of the polymer brushes. Above all of these factors, the most important was to adopt the seeding polymerization method. According to this, a small amount of the macromonomer is added to the catalyst solution, followed after 10–15 s with the addition of the remaining amount of macromonomer. This way, technically, the initiation and the propagation steps are separated in time leading to the best control over the polymerization kinetics and to reproducibility of the results. DSC analysis revealed that the crystallinity of the polymacromonomers is substantially reduced due to the architectural constraints of the structure, which prevent the organization in crystalline domains. The thermal stability of the samples and the kinetics of the thermal decomposition were studied by TGA measurements. The activation energies of the thermal decomposition were analyzed using the Ozawa–Flynn–Wall and Kissinger methodologies. The polynorbornene backbone offers increased thermal stability to the polymer brushes. Upon increasing the molecular weight of the macromonomer, the Ea values were found to decrease. The opposite effect was observed for the polymacromonomers.

## Figures and Tables

**Table 1:** Molecular characteristics of the PEO macromonomers.

Macromonomer	*M*_w_ (Da) MALDI TOF-MS	*I* = *M*_w_/*M*_n_ (SEC)
PEO 1k	1225	1.07
PEO 2k	2106	1.04
PEO 3k	3075	1.04
PEO 5k	4823	1.03

**Table 2:** Molecular characteristics of polymacromonomers synthesized following the seeding process ^a^.

PEO Macromonomer	Grubbs Catalyst	Solvent	*M*_w_, (Da) (SEC)	*M*_w_/*M*_n_	Yield (%)
1k	3rd	THF	15,000	1.15	100
1k	2nd	TOL	16,000	1.20	100
1k	3rd	TOL	25,000	1.18	100
1k	1st	TOL	47,000	1.22	100
1k	3rd	THF/CH_2_Cl_2_	56,000	1.28	100
1k	3rd	TOL	70,000	1.16	100
1k	3rd	THF	102,000	1.23	100
1k	3rd	THF	205,000	1.18	100
2k	3rd	THF/CH_2_Cl_2_	8000	1.36	100
2k	3rd	TOL	15,000	1.15	100
2k	3rd	TOL	21,000	1.13	100
2k	3rd	THF	45,000	1.22	100
3k	3rd	THF/CH_2_Cl_2_	50,000	1.40	100
3k	3rd	THF	70,000	1.32	100

^a^ Monomer concentration: 125–250 mg/mL; the polymerization was conducted at room temperature.

**Table 3:** DSC results of *α*-NBE-PEO-*ω*-OH polymacromonomers.

Sample	*M*_w_ (Da) (SEC)	*T*_M_ (°C)	Δ*H*_M_ (J/g)
1k	1000	37.76	118.20
1-15-3-THF	15,000	35.48	100.30
1-16-2-TOL	16,000	43.32	84.81
1-25-3-TOL	25,000	36.35	100.30
1-47-1-TOL	47,000	46.16	74.38
1-56-3-THF	56,000	34.95	98.42
1-70-3-TOL	70,000	45.21	102.52
1-102-3-TOL	102,000	47.61	68.62
1-205-3-TOL	205,000	47.53	79.85
2k	2000	51.42	173.10
2-15-3-TOL	15,000	47.96	129.80
2-21-3-TOL	21,000	53.40	142.10
2-45-3-THF	45,000	52.68	130.80
3k	3000	54.28	172.60
3-15-3-TOL	15,000	52.20	139.40
3-50-3-THF	50,000	49.67	137.80
5k	5000	60.10	181.40
5-245-2-TOL	245,000	57.55	141.20

**Table 4:** TGA results for sample PEO 1k.

Heating rate (°C/min)	Start (°C)	Finish (°C)	*T*_p_ (°C)	Residue % (700 °C)
3	253.26	392.50	345.88	4.9
5	248.91	394.37	358.94	4.4
7	266.32	391.26	365.77	6.8
10	276.66	383.22	361.90	3.5
15	258.24	396.23	368.88	3.6
20	261.97	404.93	383.80	4.3

**Table 5:** TGA results for sample 1-205-3-THF.

Heating rate (°C/min)	Start (°C)	Finish (°C)	*T*_p_ (°C)	Residue % (700 °C)
3	282.48	407.42	349.61	14.1
5	275.02	409.91	356.45	11.8
7	278.75	419.23	358.31	9.5
10	285.89	443.60	386.06	14.8
15	294.29	449.07	397.47	12.1
20	317.91	454.04	414.88	3.2

**Table 6:** Activation energies for the samples from the OFW method.

Weight loss (%)	*E*_a_ (J) 1-205-3-THF	*E*_a_ (J) 2-45-3-THF	*E*_a_ (J) 3-50-3-THF	*E*_a_ (J) PEO 1k	*E*_a_ (J) PEO 2k	*E*_a_ (J) PEO 3k
10	124.26	138.72	207.56	184.73	185.34	157.26
20	110.38	152.33	235.56	208.12	181.04	154.77
30	107.65	152.48	233.55	214.10	176.80	151.78
40	104.73	149.67	229.36	216.68	174.22	148.62
50	103.02	150.98	226.57	214.24	171.89	145.64
60	103.32	155.24	224.32	212.35	170.43	142.54
70	105.75	158.49	223.41	214.51	170.13	140.59
80	90.00	-	223.70	221.88	171.29	143.92
90	-	-	-	229.90	178.00	151.45

**Table 7:** Activation Energies for the polymers from the Kissinger method.

Sample	*E*_a_ (J)
1-205-3-THF	80.64
2-45-3-THF	136.82
3-50-3-THF	203.03
PEO 1K	166.72
PEO 2K	147.55
PEO 3K	122.65

## Supplementary Materials

The following are available online at www.mdpi.com/2073-4360/9/4/145/s1. Figure S1: Apparatus used for the preparation of the norbornene oxyanion in various phases of the process, Figure S2: SEC traces of PEO 2k and polymacromonomer employing Grubbs 2nd generation as the catalyst, Figure S3: SEC traces of PEO 3k and polymacromonomer employing Grubbs 2nd generation as the catalyst, Figure S4: Derivative weight loss with temperature for 2-45-3-THF under different heating rates, Figure S5: Derivative weight loss with temperature for 3-50-3-THF under different heating rates, Figure S6: OFW plots for PEO 3K, Figure S7: Kissinger plot for 1-205-3-THF, Table S1: Molecular characteristics of polymacromonomers synthesized with conventional addition method, Table S2: TGA results for sample 2-45-3-THF, Table S3: TGA results for sample 3-50-3-THF, Table S4: TGA results for sample PEO 2K, Table S5: TGA results for sample PEO 3K.
