# Supplementary material for: Synthesis, Characterization and Thermal Properties of Poly(ethylene oxide), PEO, Polymacromonomers via Anionic and Ring Opening Metathesis Polymerization
Source: Polymers (Basel). 2017 Apr 21;9(4):145. doi: 10.3390/polym9040145 (PMC6432153; doi:10.3390/polym9040145)
Supplement: Supplementary file 1 [file polymers-09-00145-s001.pdf]

polymers

Article

Synthesis, Characterization and Thermal Properties of Poly(ethylene oxide), PEO, Polymacromonomers via Anionic and Ring Opening Metathesis Polymerization

George V. Theodosopoulos [1], Christos Zisis[1], Georgios Charalambidis[2], Vasilis Nikolaou[2], Athanassios G. Coutsolelos[2], and Marinos Pitsikalis [1,*]

[1] Laboratory of Industrial Chemistry, Department of Chemistry, University of Athens, Panepistimiopolis Zografou, 15771 Athens, Greece; scimaster1821@gmail.com (G.V.T.); chriszisis1994@gmail.com (C.Z.); pitsikalis@chem.uoa.gr (M.P.)

[2] Laboratory of Bioinorganic Chemistry, Department of Chemistry, University of Crete, Voutes Campus, Heraklion 71003, Greece; gxaral@chemistry.uoc.gr (G.C.); nikolaouvasilis13@hotmail.com (V.N.); acoutsol@uoc.gr (A.G.C.)

* Correspondence: pitsikalis@chem.uoa.gr; Tel.: +003-210-727-4440

Supporting Information

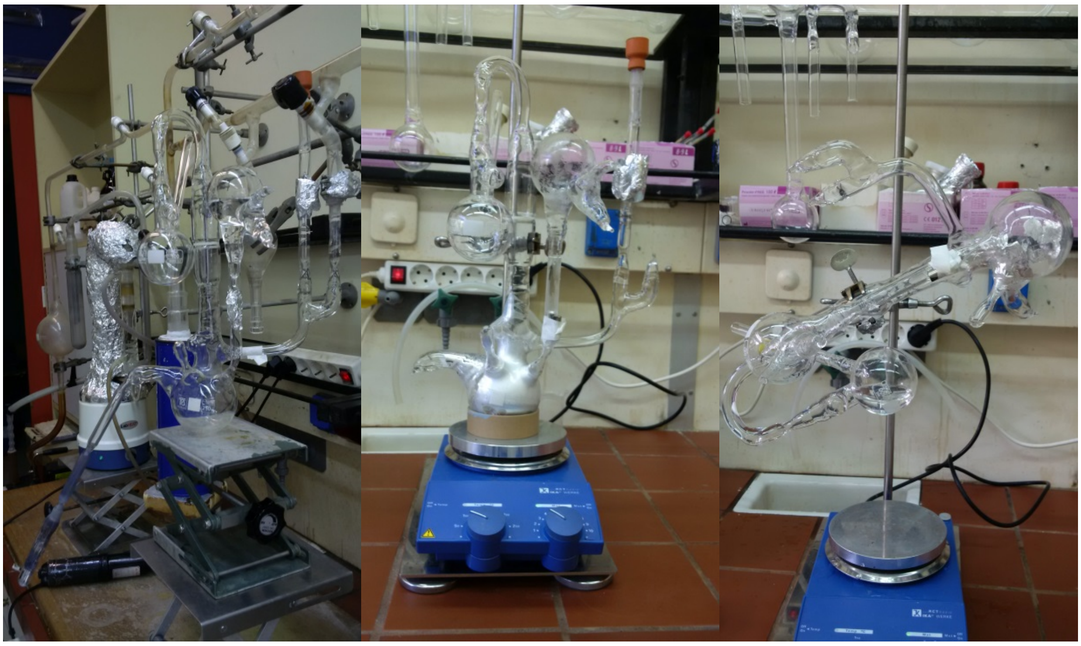

Figure S1: Apparatus used for the preparation of the norbornene oxyanion in various phases of the process

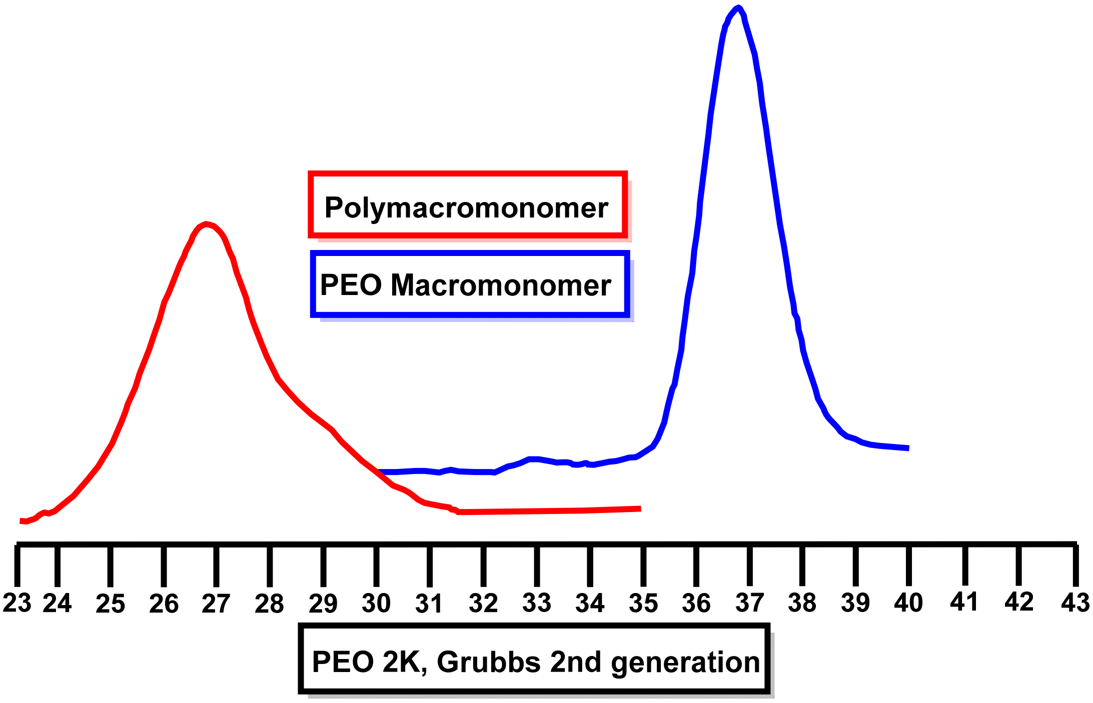

Figure S2: SEC traces of PEO 2k and polymacromonomer employing Grubbs 2nd generation as the catalyst

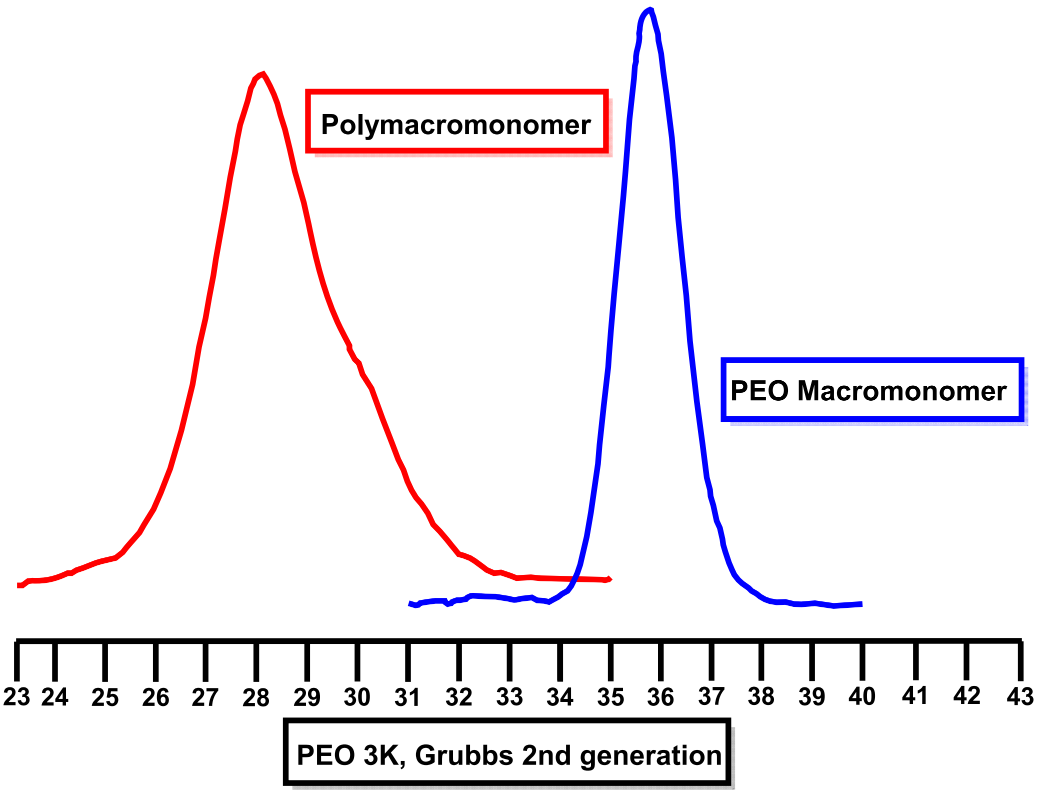

Figure S3: SEC traces of PEO 3k and polymacromonomer employing Grubbs 2nd generation as the catalyst

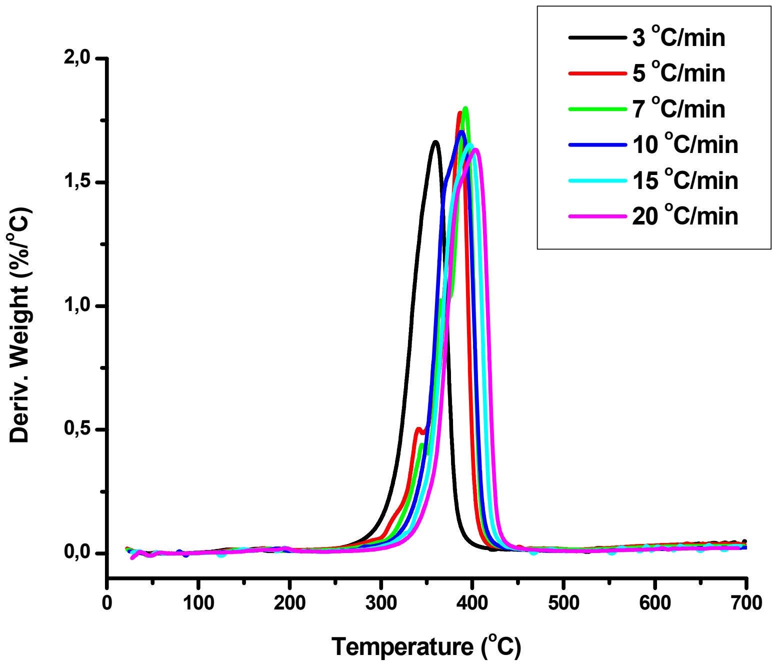

Figure S4: Derivative weight loss with temperature for 2-45-3-THF under different heating rates

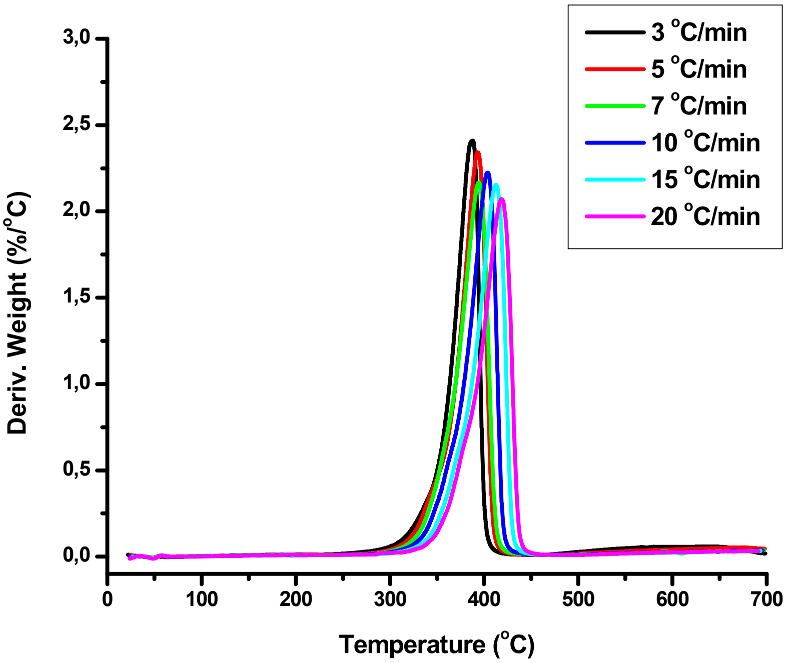

Figure S5: Derivative weight loss with temperature for 3-50-3-THF under different heating rates

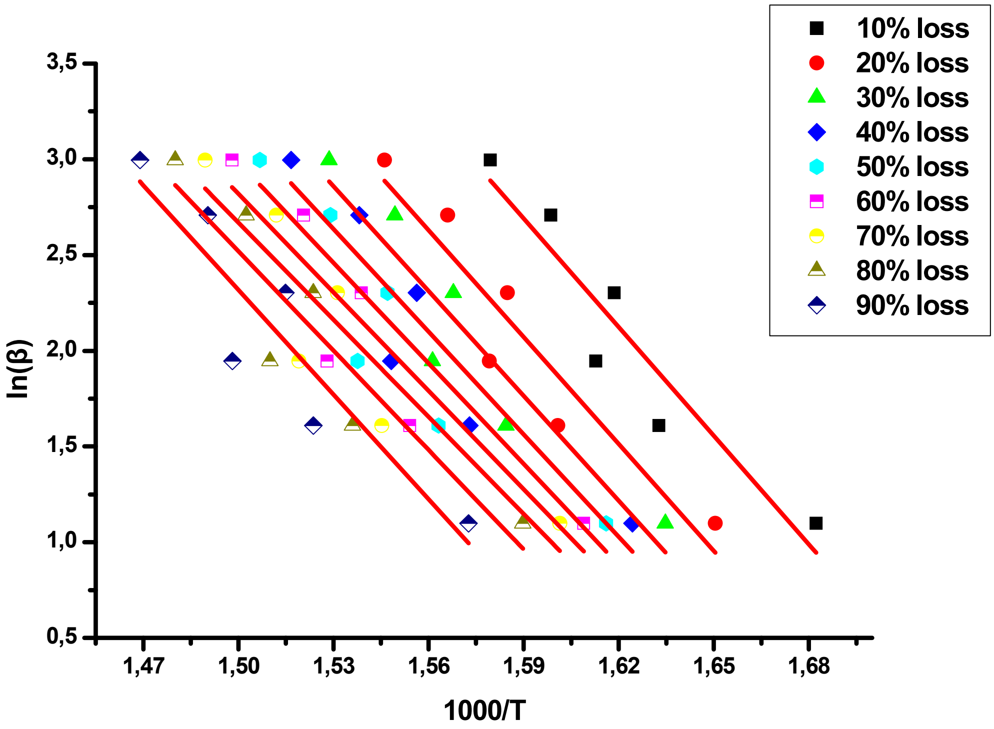

Figure S6: OFW plots for PEO 3K

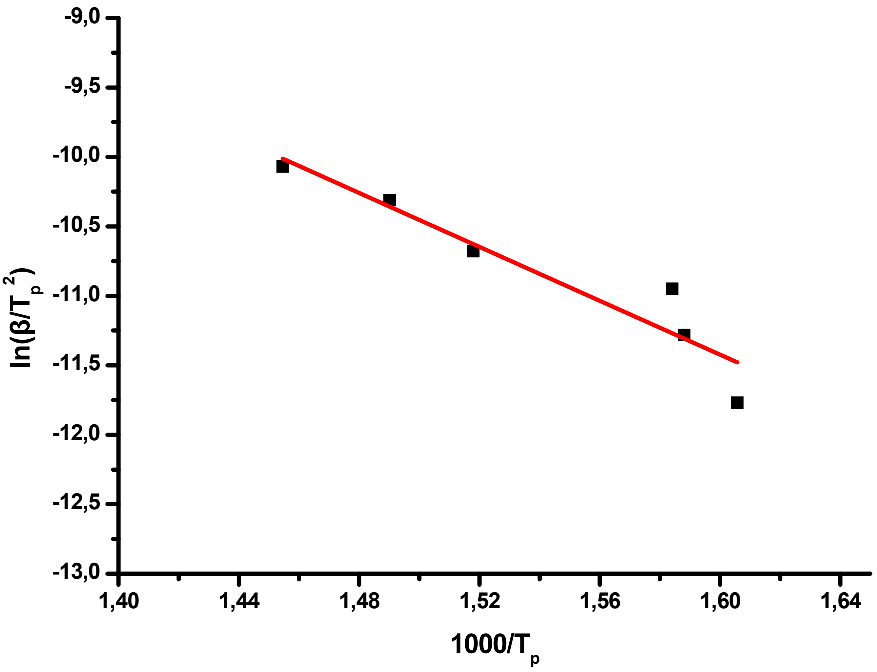

Figure S7: Kissinger plot for 1-205-3-THF

Table S1: Molecular characteristics of polymacromonomers synthesized with conventional addition method

PEO macro/mer	Grubbs catalyst	Solvent	M_w (SEC)	M_w/M_n	yield (%)
1k	2nd	TOL	85,000	1.29	100
1k	2nd	TOL	50,000	1.09	100
1k	2nd	TOL	10,000	1.09	100
2k	2nd	TOL	128,000	1.50	100
2k	2nd	TOL	80,000	1.30	100
3k	2nd	TOL	70,000	1.32	100
3k	2nd	TOL	90,000	1.25	100
5k	2nd	TOL	95,000	1.45	98
5k	2nd	DCBz	100,000	Bimodal1.37	93
5k	2nd	DCBz	120,000	Bimodal1.30	98
5k	2nd	DCBz	160,000	1.63	100

5k	2nd	CH_2Cl_2	75,000	Bimodal1.80	35
5k	2nd	EtOH/CH_2Cl_2	40,000	1.16	10
5k	2nd	EtOH/CH_2Cl_2	245,000	1.28	70

Table S2: TGA results for sample 2-45-3-THF

Heating Rate (°C/min)	Start (°C)	Finish (°C)	T_p (°C)	Residue % (700 °C)
**3**	269.43	404.93	360.18	12.7
**5**	295.53	419.23	386.29	17.4
**7**	298.64	427.93	392.50	16.8
**10**	297.26	434.37	387.48	10.7
**15**	316.05	439.74	397.47	10.1
**20**	313.56	445.34	403.07	9.5

Table S3: TGA results for sample 3-50-3-THF

Heating Rate (°C/min)	Start (°C)	Finish (°C)	T_p (°C)	Residue % (700 °C)
**3**	295.53	421.10	387.53	2.4
**5**	300.51	424.20	393.75	2.4
**7**	303.61	427.93	394.37	6.2
**10**	306.10	436.64	403.07	4.9
**15**	316.56	447.20	412.39	4.8
**20**	318.53	451.55	417.99	4.1

Table S4: TGA results for sample PEO 2K

Heating Rate (^oC/min)	Start (^oC)	Finish (^oC)	T_p (^oC)	Residue % (700 ^oC)
**3**	252.64	384.42	350.85	4.2
**5**	262.59	394.37	362.04	3.9
**7**	260.72	394.37	357.69	2.3
**10**	273.10	389.61	359.77	1.3
**15**	283.72	409.29	381.93	2.3
**20**	271.29	410.53	383.80	2.4

Table S5: TGA results for sample PEO 3K

Heating Rate (^oC/min)	Start (^oC)	Finish (^oC)	T_p (^oC)	Residue % (700 ^oC)
**3**	255.75	390.02	347.13	0.8
**5**	260.72	404.93	371.37	2.6
**7**	264.45	413.64	383.80	2.6
**10**	281.24	408.04	379.45	1.3
**15**	279.37	422.34	383.18	1.6
**20**	286.21	429.18	394.37	2.1
